# Effectiveness of smartphone-based ambulatory assessment (SBAA-BD) including a predicting system for upcoming episodes in the long-term treatment of patients with bipolar disorders: study protocol for a randomized controlled single-blind trial

**DOI:** 10.1186/s12888-018-1929-y

**Published:** 2018-10-26

**Authors:** Esther Mühlbauer, Michael Bauer, Ulrich Ebner-Priemer, Philipp Ritter, Holger Hill, Fabrice Beier, Nikolaus Kleindienst, Emanuel Severus

**Affiliations:** 1 Department of Psychiatry and Psychotherapy, University Medical Center Dresden, Dresden, Germany; 2 Department of Sport and Sport Science and House of Competence, Karlsruhe Institute of Technology, Karlsruhe, Germany; 3 Central Institute of Mental Health, Institute for Psychiatric and Psychosomatic Psychotherapy, Mannheim, Germany

**Keywords:** Bipolar disorder, Ambulatory assessment, Prevention, Early warning signs

## Abstract

**Background:**

The detection of early warning signs is essential in the long-term treatment of bipolar disorders. However, in bipolar patients’ daily life and outpatient treatment the assessment of upcoming state changes faces several difficulties. In this trial, we examine the effectiveness of a smartphone based automated feedback about ambulatory assessed early warning signs in prolonging states of euthymia and therefore preventing hospitalization. This study aims to assess, whether patients experience longer episodes of euthymia, when their treating psychiatrists receive automated feedback about changes in communication and activity. With this additional information an intervention at an earlier stage in the development of mania or depression could be facilitated. We expect that the amount of time will be longer between affective episodes in the intervention group.

**Methods/design:**

The current study is designed as a randomized, multi-center, observer-blind, active-control, parallel group trial within a nationwide research project on the topic of innovative methods for diagnostics, prevention and interventions of bipolar disorders. One hundred and twenty patients with bipolar disorder will be randomly assigned to (1) the experimental group with included automated feedback or (2) the control group without feedback. During the intervention phase, the psychopathologic state of all participants is assessed every four weeks over 18 months. Kaplan-Meier estimators will be used for estimating the survival functions, a Log-Rank test will be used to formally compare time to a new episode across treatment groups. An intention-to-treat analysis will include data from all randomized patients.

**Discussion:**

This article describes the design of a clinical trial investigating the effectiveness of a smartphone-based feedback loop. This feedback loop is meant to elicit early interventions at the detection of warning signs for the prevention of affective episodes in bipolar patients. This approach will hopefully improve the chances of a timely intervention helping patients to keep a balanced mood for longer periods of time. In detail, if our hypothesis can be confirmed, clinical practice treating psychiatrists will be enabled to react quickly when changes are automatically detected. Therefore, outpatients would receive an even more individually tailored treatment concerning time and frequency of doctor’s appointments.

**Trial registration:**

ClinicalTrials.gov: NCT02782910: Title: “Smartphone-based Ambulatory Assessment of Early Warning Signs (BipoLife_A3)”. Registered May 25 2016.

Protocol Amendment Number: 03. Issue Date: 26 March 2018. Author(s): ES.

## Background

Bipolar disorder as an affective illness with chronic features shows a very diverse and often unpredictable course of illness concerning the number, length and frequency of recurrent depressive and (hypo) manic states [1]. This condition causes high costs both from an individual as well as from a societal point of view [2]. Patients with bipolar disorder suffer from marked impairment in everyday social and occupational life, e.g. experiencing a diminished quality of life, often due to lower levels of psychosocial functioning [3, 4]. Furthermore, outpatient and inpatient treatment of affective episodes leads to high health care costs. Necessary reductions of working hours, higher absenteeism or premature pension are common and cause substantial secondary economic costs [5, 6].

Though not yet proven the detection of early warning signs (EWS) is considered to improve the course of bipolar illness [7] but it is mainly the patient’s own responsibility as he or she is usually seen by the treating psychiatrist once every few weeks or even months, especially when euthymic. Mood charting as the common tool for EWS detection has several limitations in clinical practice. Firstly, even if done on a daily basis, mood-charting as retrospective and subjective self-assessment, is generally prone to be affected or biased by heuristics, cognitive impairment and symptoms of an evolving episode [8, 9]. Also, demographic characteristics may interfere with the person’s capacity to precisely report the items of interest [10]. Furthermore, the frequency of recording mood, sleep and medication intake reduces over time as many patients with bipolar disorder get tired of mood charting, especially in long episodes of euthymia. However, as the course of bipolar illness remains highly unpredictable even after long periods of euthymia, the cessation of mood charting may turn out to be detrimental [11].

To overcome the outlined problems, ambulatory monitoring has recently been implemented in the long-term treatment of bipolar disorders. This approach is beneficial as it delivers objective, real-time and real-life data in an instant. As a result, much research has been accumulated over the past couple of years employing new technologies [12–18]. Software-based mood charting using the ChronoRecord software is advantageous compared to paper-pencil methods regarding data storage and legibility. This has been validated both for depressive and manic symptoms in bipolar patients [12, 13]. To the best of our knowledge, there are published results of only one clinical trial using smartphones to predict and prevent upcoming episodes. This preliminary single-blind randomized trial studied the impact of electronic self-monitoring of subjective and objective symptoms of bipolar disorders on changes in affective symptoms (primary outcome) as well as depressive and manic symptoms. It was initiated in September 2011 and is referred to as the MONARCA trial [14]. According to reported results of the MONARCA trial electronic self-monitoring, via smartphone, had no significant effect on bipolar symptomatology. For patients in the intervention group there was even a trend of more sustained depressive symptoms [19]. In a second trial the researchers aimed to investigate the usefulness of automated feedback on objective and subjective smartphone data for the reduction of affective symptoms [20]. These results have not yet been published.

Our clinical trial is the first one in German speaking countries to investigate the usefulness of a smartphone-based feedback loop triggering early interventions at the detection of warning signs for the prevention of severe depressive and manic states in bipolar patients. The objective of this randomized controlled trial, referred to as the SBAA-BD study, is to assess the effectiveness of automated feedback about continuous smartphone based ambulatory monitoring of potential early warning signs of upcoming depressive/(hypo) manic episodes. The study investigates whether time to new affective episodes and following hospitalizations can be prolonged in the intervention group. In the MONARCA I trial the placebo group were given a cell phone without an application to monitor the symptoms of interest. However, the knowledge of being continuously monitored for early warning signs may have an impact on the outcome(s) in mood disorders [21]. For this reason we decided to monitor early warning signs in the control group in an identical fashion, by smartphone-based ambulatory assessment including real-time data capture (SBAA). Patients are informed about the group that they are assigned to. Therefore within the 18 months intervention phase identical ambulatory monitoring of EWS of a depressive or (hypo) manic episode is taking place in the control group, serving as a comparator. However the treating psychiatrist has no insight in potential deviations of the patients’ smartphone data from his or her individually calculated baseline data. Irrespective of group assignment, all patients receive guideline-based, state-of-the-art maintenance treatment. In comparison to the MONARCA I trial, the parameters assessed in our study originate exclusively from objective data captured in real time. No event- or time-triggered subjective data like mood or energy level have to be entered by the participants so that the probability of subjective and retrospective biases is expected to be reduced and adherence to study protocol might be enhanced. Additionally, the possibility of worsening depressive symptoms could be reduced as patients do not have to enter their subsequent depressive symptoms every day and process their current mental state in a negative manner [19]. Additionally, patients in this trial stay enrolled for a longer period of time (18 months) than in most of the trials mentioned above. Additionally, we are pursuing a larger sample with 120 randomized patients to overcome limitations of previous work on smartphone-based objective monitoring in bipolar disorder [22]. All data is automatically recorded at the very moment when the behavior (communication and activity) occurred without driving the patients’ attention to the fact that his or her behavior is currently monitored.

We hypothesize that the length of time to a new affective episode and subsequently necessary hospitalization is significantly longer in the Smartphone-Based Ambulatory Assessment group (SBAA+). In this condition clinicians receive automated feedback about substantial change from baseline concerning their patients’ objective smartphone usage data (communication and activity patterns). As a consequence they can intervene at an earlier stage in the development of mania or depression, compared to the Smartphone-Based Ambulatory Assessment group, excluding personalized real-time data-driven therapeutic interventions (SBAA).

## Methods/design

### Design and study setting

This study is a randomized, multi-center, rater-blinded, active-control, parallel group trial within a nationwide research project on the topic of innovative methods for diagnostics, prevention and interventions of bipolar disorders [23, 24]. Assessments are scheduled weekly in the prerandomization phase (4–20 weeks) and monthly in the intervention phase (72 weeks). End of intervention phase is followed by a post-intervention visit after four weeks. Five outpatient centers of psychiatric clinics at German university hospitals (Dresden, Berlin, Hamburg-Eppendorf, Frankfurt, Bochum), specialized in the treatment of bipolar disorders will assess data for this trial. Recruitment shortages are planned to be overcome by opening further study sites (Neuruppin, München, Tübingen). The coordinating site is the University Medical Center in Dresden, supported by the Coordination Center for Clinical Trials (KKS). The clinical trial conforms to the requirements of the MDR (Medical Device Regulation).

### Participants

Potentially eligible participants will be approached at the participating university hospitals (outpatient clinic, inpatient units, and day clinic) by their treating psychiatrists. Furthermore, our study project was presented in local print media. In addition, potential participants could find relevant information on the trials homepage [24]. Finally, board certified psychiatrists were provided with the necessary information by mail as well as in the context of continued medical education training at our hospital. Eligible participants for this trial meet diagnostic criteria for Bipolar Disorders I or II and they can be either symptomatic or asymptomatic at time of enrollment (DSM-5: 296.4×; 296.5×; 296.89). At time of randomization, participants have to be remitted (DSM-5: 296.45, 296.46; 296.55, 296.56; 296.89). Further inclusion criteria consist of (1) age > 18 years, (2) signed informed consent prior to enrollment, (3) ≥ 3 affective episodes in last 5 years, one of them being a (hypo) manic episode, (4) smartphone usage, (5) and each patient must have a sufficient level of understanding to agree to all tasks required by the protocol. Exclusion criteria are comprised of (1) participation in another clinical intervention trial for the last 4 weeks prior to enrollment, (2) substance or other disorders that prevent the patient to understand the nature and consequences of the clinical trial, (3) signs that the patient will probably not comply with the study protocol (e.g. lack of cooperativeness), (4) current substance use disorder (except for tobacco and caffeine), moderate or severe, at enrollment, (5) borderline personality disorder, antisocial personality disorder, (6) dementia, organic brain disorders, (7) patients with a physical illness that probably requires inpatient treatment for more than 4 weeks per annum (exclusion: bipolar disorder), (8) patients working in a shift system which does not ensure regular night sleep, (9) relatives or persons in relation of dependence to study personnel, (10) women of childbearing age, excluding women meeting the following criteria: post-menopausal, postoperative (6 weeks after ovariectomy), regular and correct use of a contraceptive method, sexual abstinence or vasectomy of partner, (11) pregnancy or nursing.

The study population of aim, who meet our criteria, is representative of bipolar patients in outpatient treatment. Pregnant and nursing women as well as women who might get pregnant during the trial are excluded as a requirement for trials conforming to the Medical Device Regulation (MDR).

### Intervention

All patients included in this study use a smartphone with an installed application called movisensXS. MovisensXS is an electronic e-diary software, which additionally can track and transmit smartphone data collected from sensors inside the device. Patients can decide, whether they want to use the study smartphone (a currently available mid-priced Samsung smartphone) or install the app via Google Play Store on their own device. Technical requirements include Android version 4.0 or higher, CPU of 600 MHz or above, acceleration sensor and GPS. In this study, the movisensXS app monitors current location via GPS, an estimate of the covered steps (using the devices’ acceleration sensor), mobile communication parameters (number and duration of incoming and outgoing calls, number and length of incoming and outgoing text messages, number of different call and text contacts), usage duration of common communication apps (e.g. WhatsApp, Facebook Messenger), number and duration of times the display of the device is on/off, as well as rates of transmitted and received data. For enrolled patients, a pseudonymized electronic file is created on the movisensXS platform and all data is transmitted daily to a secured server. Data protection requirements are complied with at any stage of data storage and transmission processes. Every 24 h data are drawn from the data storage server by a feedback server via a secure connection and data of patients in the intervention group are compared with individual baseline data performed. The intervention-algorithm is based on a combination of threshold values associated with parameters which are linked to DSM-5 criteria of bipolar disorders, i.e. change in goal-directed or non-goal directed activity and change of communication pattern (e.g. more talkative than usual). Psychomotor agitation/retardation is operationalized by movement patterns derived from two smartphone sensor systems. Daily travel distances in kilometers per day are calculated via GPS coordinates. The acceleration sensor in the smartphone tracks acceleration in three spaces. Daily movement activity is assessed by number of steps. General inactivity is calculated by numbers of hours without any smartphone activity, which includes no acceleration data, no phone calls or text messages and the smartphones display being turned off. Communication is operationalized by tracking the frequency and length of phone calls and text messages using the smartphone.

**Fig. 1:**
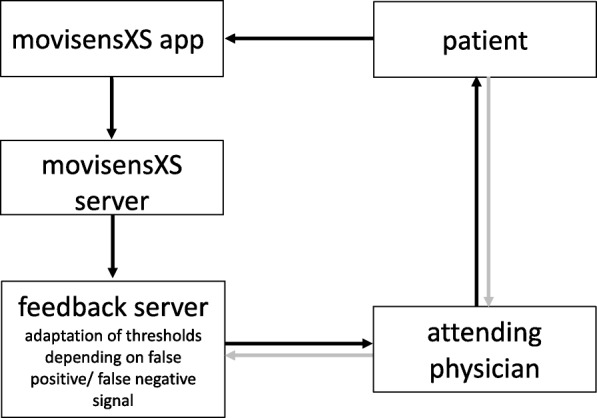
Information flow.  standard information flow  feedback information flow

First, daily values are calculated for all monitored parameters. During a stabilization period (four weeks of euthymia) in the prerandomization phase individual baseline values (mean and standard deviation) for the persons’ typical smartphone usage, communication and activity behavior in the following eight parameters are calculated: (1) travelled distance (km/day), (2) steps/day, (3) number of outgoing calls/day, (4) average phone call duration/day, (5) length of outgoing text messages/day, (6) hours when smartphone is inactive, (7) frequency of display on/day and (8) duration of display on/day. During the intervention phase daily deviations of daily values from baseline are calculated. A defined alarm threshold is exceeded when two or more parameters exceed or fall below the 20% or above the 80% percentile on three subsequent days. The direction of the deviation has to be the same for each variable on these three days but can differ between different variables. For patients in the intervention group, alarms are automatically forwarded by email to the respective study center. The attending psychiatrist contacts the patient and decides whether an affective episode is present or developing and carries out the next standard procedures as in usual practice, e.g. appointing a personal contact to discuss treatment options with the patient. If no affective episode is present and the alarm is not explicable by current external circumstances of the patients’ situation the signal is rated as a false alarm. In this case, the alarm thresholds are raised by 5% (values of the 20% and the 80% percentile). In case of an undetected mood episode during the intervention phase the thresholds will be lowered by 10%. Information flow is depicted in Fig. 1.

To prevent false alarms during e.g. holidays or physical illness, patients can individually pause the monitoring of data during these episodes. For patients in the control group, no alarms will be forwarded to the respective study center. All patients receive guideline-based, state-of-the-art maintenance treatment for bipolar disorder. In addition, within the study protocol requirements the investigators aim at giving maximum flexibility to the study participants as to the required study appointments. To ensure patients adherence and continued data collection in both groups, an automated email is sent to the study center when no smartphone activity is monitored within 24 h. In case of continued non-adherence to study protocol, such as repeatedly deactivated smartphone for more than 24 h, patients can be excluded from further participation. To improve adherence, patients receive an expense allowance of 15 € per month to ensure that costs are covered for possible necessary adaptions of mobile plans. If using the study smartphone, patients can further keep the device once participation is completed following study protocol so that changing back to another device after study participation is excluded as a recruitment hindrance. All patients can withdraw previously provided consent at any time without specification of reasons and without any adverse or disadvantageous consequences.

### Measures

#### Diagnostic and rating scales

At screening, bipolar diagnosis is assessed with the Structured Clinical Interview for DSM Disorders (SCID-I, German version) [25] for DSM-IV. An additional question concerning abnormally and persistently increased activity or energy is included to conform to current DSM-5 criteria for depressive, manic or hypomanic episodes [26]. The semi-structured interview is conducted face-to-face and is established for diagnosing mental disorders. Throughout the study, current affective episodes are assessed with the SCID-I section A for affective episodes, including the additional DSM-5 criterion described above. Furthermore, (hypo) manic symptoms are dimensionally rated with the Young Mania Rating Scale (YMRS) [27] which consists of 11 items. Symptom severity is rated with 0 to 4 points; four items are double-weighted. A total value below 12 is considered clinically insignificant. The YMRS is regarded as gold standard in mania psychometry and its reliability (*r* = .93) and validity (*r* = .86 to .89) are well studied [27]. Depressive symptoms are dimensionally rated with the Inventory of Depressive Symptomatology, clinician version (IDS-C 30) [28] which consists of 30 items. Symptom severity is rated with 0 to 3 points. A total value below 18 is considered clinically insignificant. The IDS is well established in the assessment of depressive symptom severity. Good reliability (*r* = .89) and validity (*r* = .81) are reported for patients with bipolar disorder [29]. All ratings will be performed by trained and experienced psychologists and/or psychiatrists and data is directly entered in the electronic database. In addition, source data are documented by paper and pencil and signed by the investigator upon completion. Paper versions of all instruments used are provided in the investigator site file in case a direct entry of data should not be possible due to technical reasons.

#### Outcomes

Primary outcome of this trial is time to new affective episode, defined by DSM-5 criteria for depressive, manic or hypomanic episode. The primary outcome will be determined by SCID I section A (for DSM-IV, additional DSM-5 question for energy/activity being included), criteria for current depressive and (hypo) manic episode, in monthly assessments during the intervention phase. The primary outcome is clinically highly relevant to determine the affective and mental stability of bipolar patients.

Secondary endpoints are (1) time to a new depressive episode, (2) time to a new (hypo) manic episode, (3) time to a new affective episode defined by DSM-5 but with modified time criterion of ≥ four days of episode duration, (4) percentage of assessments during intervention phase at which the original DSM-5 criteria for an affective episode are met, (5) percentage of assessments during intervention phase at which the modified DSM-5 criteria for an affective episode are met. These outcomes will also be determined by SCID I section A (for DSM-IV, additional DSM-5 question for energy/activity being included), criteria for current depressive and (hypo) manic episode, in monthly assessments during the intervention phase. Further secondary outcomes are (6) percentage of assessments during intervention phase at which criteria for an affective episode, defined by YMRS (> 12) and IDS-30 (> 18) score are met and (7) average severity of manic and depressive symptoms at assessments during intervention phase. These criteria are estimated by total YMRS and IDS-C30 scores at every assessment. Operationalization of the secondary outcomes (8) percentage of assessments during intervention phase at which the patient was hospitalized for an affective episode, and (9) percentage of days during intervention phase at which the patient was hospitalized for an affective episode, is self-explanatory. (10) Number of adverse events is measured by the SAE (serious adverse event) reporting form included in the electronic case report form (eCRF).

The outcome measures chosen in our trial are commonly used in similar long-term treatment trials in bipolar disorders. In particular our primary endpoint, time to new episode, is the most established endpoint in new approval-seeking trials for psychopharmacological drugs in long-term treatment of bipolar disorders. In addition, our secondary endpoints will allow a better insight in the robustness of our primary endpoint and in developing an understanding of the economic impact of our intervention.

### Study procedure

Inpatients and outpatients with bipolar disorder I/II (currently symptomatic or stable) are assessed for eligibility by experienced clinical staff working at the study site who will present all relevant information about the background, design, procedure, etc. of the study to the patient and obtain informed consent in case of given eligibility. After having signed informed consent, a pseudonymized file is created for the patient in the electronic database and the movisensXS smartphone application is installed on a study smartphone handed over to the patient for his or her time of study participation, or, if wished, on the patient’s own device. Participants continuously receive open, guideline-based treatment for potential current (hypo)manic and depressive symptomatology for up to 20 weeks at the study sites outpatient clinics. As soon as stabilisation criteria are met (Young Mania Rating Scale total score < 12 and Inventory of Depressive Symptomatology, clinician version total score < 18) during four consecutive weeks, a baseline of the persons individual smartphone usage pattern during euthymia is automatically defined. Having fulfilled randomization criteria (four consecutive weeks with YMRS total score < 12 and IDS-C30 total score < 18), patients are randomly assigned to the Smartphone-Based Ambulatory Assessment group, including real-time data capture and data-driven therapeutic interventions (SBAA+), or to the Smartphone-Based Ambulatory Assessment group, only including real-time data capture (SBAA).

### Randomization and blinding

A randomization list is stored in the electronic database where all collected data is entered using eCRFs. Based on this list, patients are allocated to the two groups (SBAA resp. SBAA+) by block randomization, stratified by age and type of bipolar disorder (I or II) for every study site. Pseudonymized files are created in the database for enrolled patients. The system allocates patients to the groups corresponding to the stored algorithm for randomization and the required stratification factors. The patient and the assigned psychiatrist are automatically and instantly informed about which group (SBAA vs. SBAA+) the patient was allocated to once randomization is carried out via the database system. As regular ratings must not be biased by group allocation knowledge, the observer/rater remains blinded. Both treating psychiatrist and patient are asked explicitly not to disclose any information allowing conclusions regarding group allocation. As the study psychiatrists are not blinded, there was no need to provide special procedures for emergency unblinding.

The intervention phase is planned for 18 months (72 weeks). During this time, state-of-the-art treatment of the bipolar condition continues for both groups. Attending psychiatrists will receive automatic feedback from the movisensXS feedback server, every time the previously calculated individual thresholds of patients in the SBAA+ group are significantly exceeded in any direction. A standardized procedure follows, where the psychiatrist contacts the patient to assess, whether (a) the changes in smartphone usage patterns can be explained by external circumstances, or (b) show no hint for the beginning of a new affective episode (false alarm), or (c) the changes result from early signs of bipolar episodes. In this case treatment options are discussed with the patient as in regular guideline-based treatment. Additionally, in the case that the rater detects an affective episode, the study clinician will be informed so that thresholds can be changed towards more sensitivity if the patient is enrolled in the intervention group and no automatic alarm has been present.

**Fig. 2:**
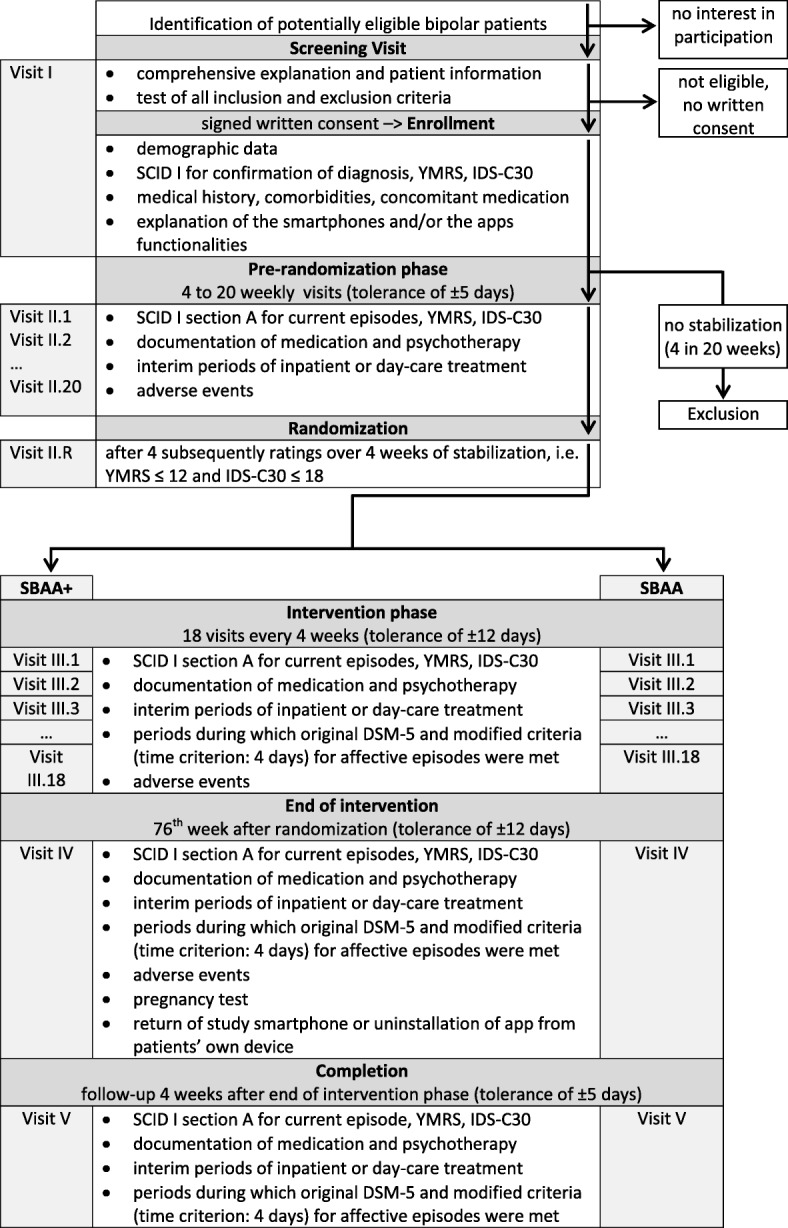
Study flow

On a monthly base, assessments of bipolar psychopathology (SCID, YMRS, IDS-C30) are conducted by the rater who is blind to the patients’ group assignment. A post-trial follow-up of four weeks per patient follows subsequently after the end of the 72 weeks intervention phase. Figure 2 shows the schematic study flow, serving as a detailed overview for the time schedule of enrolment, intervention, assessment and visits for the participants.

The study population participants should be recruited during 18 months, the first patient was enrolled in January 2017.

### Statistical analysis

#### Confirmatory analysis

The primary objective (i.e. comparison of time to a new episode under SBAA+ vs SBAA) will be addressed with survival analysis. Kaplan-Meier plots will be used for estimating the survival functions; a Log-Rank test will be used to formally compare time to a new episode across treatment groups. By using this strategy, the analysis will include data from all patients who were randomized (ITT analysis). Although randomization will be used to attain comparable conditions at baseline in both groups, imbalances with respect to major confounders cannot be completely ruled out. In this case, the predefined survival analyses will be backed up by a Cox-regression which includes these confounders. All patients who have been randomized will be included in the statistical analyses.

#### Explorative analysis

Regarding secondary endpoints, separate survival analyses will be applied according to the variables level of measurement. For continuous endpoints, means and standard deviations respectively medians and quartiles are applied (graphically according to confidence intervals respectively boxplots), for categorical endpoints absolute and relative frequency are applied. Depending on the distribution of the analyzed variables, a *t*-test, *U*-test and *Chi*^2^-test are applied. For intraindividual comparisons a paired *t*-test is applied for normally distributed variables, and a Wilcoxon-test for not normally distributed variables.

Access to the final trial dataset is granted to the sponsor who is the owner of all data. Access is also granted to biometry staff.

### Sample size calculation

Required sample size was calculated as in: *n* = 360 to be assessed for eligibility; *n* = 180 to be allocated to trial; *n* = 120 to be randomised to SBAA+ or SBAA; *n* = 120 to be analysed. The sample size of *n* = 60 per group was based on a power calculation for survival analysis with constant hazard and drop-out rates. This power calculation requires specific assumptions regarding (a) drop-out rate in SBAA (placebo), (b) global drop-out rate and (c) hazard ratio. Drop-out rate in SBAA (Placebo) was calculated with data from the psychiatric outpatient clinic, as this population most closely corresponds to the examined sample. Accordingly we expect a monthly hazard rate of 6%, which is equivalent to a relapse rate of 62% for the course of the trial. From our experience with comparable patients in similar studies we expect a drop-out rate of approximately 2.5%. This is equivalent to a drop-out rate of 26% for the course of the trial. A hazard ratio of 0.5 is estimated, according to a reduction of the relapse rate from 62 to 41% in the intervention group. This assumption is consistent with results of a Cochrane review on interventions, helping patients to identify early warning signs. The hazard ratio of 0.5 equals the median hazard ratio in studies, which compare TAU and TAU plus detection of EWS for relapse. This estimation is considered rather conservative as smartphone usage in this trial expectedly leads to a quicker and more reliable detection of warning signs, thus we expect a greater effect compared to the studies mentioned. Hence, with 81% this trial is sufficiently powered to demonstrate the advantage of SBAA+ compared to SBAA regarding the primary outcome (α = .05, one-tailed). Power calculation was performed using SAS software, version 9.3 (2011, Cary, NC).

## Discussion

This article describes the design of a clinical trial investigating the effectiveness of a smartphone based feedback loop eliciting early interventions at the detection of warning signs for the prevention of depressive and manic states in bipolar patients. As a result of this trial and the data obtained new passive, non-invasive monitoring solutions will be better supported in the future. The medium-term objective is the development of applications which help optimize the timing of suitable interventions in bipolar patients without substantial active involvement of the patients themselves. This approach will hopefully improve the chances of a timely intervention that helps patients to keep a balanced mood for longer periods of time as well as contribute to a higher level of functioning and quality of life.

### Strengths and limitations

This study uses new and widely spread smartphone technology for the further improvement of the long term treatment of patients with bipolar disorder. Using a smartphone application to monitor everyday communication and activity could operationalize changes in affective states. This approach is non-invasive, requires patients’ minimal effort and, if proven effective, will be a cost-effective and promising solution to help people with bipolar disorder to spend less time in affective episodes. With a total duration of 21 months the presented study comprises a comparatively long period of time of continuous ambulatory assessment. The 18-month intervention phase allows for a strong data basis for analyzing the effectiveness of the described feedback algorithm. Limitations of this trial might concern the representability of the sample, as some patients cannot participate. This includes patients who want to use their own iPhone (movisensXS only available for Android) and women with an acute desire to have children. Due to technical limitations, use duration of common communication apps (WhatsApp, Facebook Messenger …) could not be monitored from the beginning of the trial and therefore this data is not included in the feedback algorithm.

### Trial status

Recruitment and enrollment of the first patient started in January 2017.

## Abbreviations

CPU: Central Processing Unit
DSM: Diagnostic and Statistical Manual of Mental Disorders
DSMB: Data monitoring and safety committee
eCRF: Electronic case report form
EWS: Early warning signs
GPS: Global Positioning System
IDS-C 30: Inventory of Depressive Symptomatology, clinician version
ITT: Intention to treat
KKS: Coordination Center for Clinical Trials
MDR: Medical Device Regulation
SAE: Serious adverse event
SBAA: Smartphone-Based Ambulatory Assessment
SCID-I: Structured Clinical Interview for DSM Disorders
TAU: Treatment as usual
YMRS: Young Mania Rating Scale
